# Iatrogenic Alzheimer’s disease in recipients of cadaveric pituitary-derived growth hormone

**DOI:** 10.1038/s41591-023-02729-2

**Published:** 2024-01-29

**Authors:** Gargi Banerjee, Simon F. Farmer, Harpreet Hyare, Zane Jaunmuktane, Simon Mead, Natalie S. Ryan, Jonathan M. Schott, David J. Werring, Peter Rudge, John Collinge

**Affiliations:** 1 MRC Prion Unit at UCL and UCL Institute of Prion Diseases, London, UK; 2 National Prion Clinic, National Hospital for Neurology and Neurosurgery, London, UK; 3 Department of Neurology, National Hospital for Neurology and Neurosurgery, London, UK; 4 UCL Queen Square Institute of Neurology, London, UK; 5 Lysholm Department of Neuroradiology, National Hospital for Neurology and Neurosurgery, London, UK; 6 Department of Clinical and Movement Neurosciences and Queen Square Brain Bank for Neurological Disorders, UCL Queen Square Institute of Neurology, London, UK; 7 Division of Neuropathology, National Hospital for Neurology and Neurosurgery, London, UK; 8 Department of Neurodegenerative Disease, Dementia Research Centre, UCL Queen Square Institute of Neurology, London, UK; 9 UK Dementia Research Institute at UCL, London, UK; 10 Stroke Research Centre, UCL Queen Square Institute of Neurology, London, UK; 11 Stroke Service, National Hospital for Neurology and Neurosurgery, London, UK

**Keywords:** Alzheimer's disease, Alzheimer's disease

## Abstract

Alzheimer’s disease (AD) is characterized pathologically by amyloid-beta (Aβ) deposition in brain parenchyma and blood vessels (as cerebral amyloid angiopathy (CAA)) and by neurofibrillary tangles of hyperphosphorylated tau. Compelling genetic and biomarker evidence supports Aβ as the root cause of AD. We previously reported human transmission of Aβ pathology and CAA in relatively young adults who had died of iatrogenic Creutzfeldt–Jakob disease (iCJD) after childhood treatment with cadaver-derived pituitary growth hormone (c-hGH) contaminated with both CJD prions and Aβ seeds. This raised the possibility that c-hGH recipients who did not die from iCJD may eventually develop AD. Here we describe recipients who developed dementia and biomarker changes within the phenotypic spectrum of AD, suggesting that AD, like CJD, has environmentally acquired (iatrogenic) forms as well as late-onset sporadic and early-onset inherited forms. Although iatrogenic AD may be rare, and there is no suggestion that Aβ can be transmitted between individuals in activities of daily life, its recognition emphasizes the need to review measures to prevent accidental transmissions via other medical and surgical procedures. As propagating Aβ assemblies may exhibit structural diversity akin to conventional prions, it is possible that therapeutic strategies targeting disease-related assemblies may lead to selection of minor components and development of resistance.

## Abstract (web summary)

A small number of patients who received growth hormone preparations contaminated with seeds of the amyloid-beta protein developed Alzheimer’s disease many years after treatment.

## Main

Mammalian prions are protein-only infectious agents that cause fatal neurodegenerative diseases^1^. They comprise assemblies of misfolded host-encoded cellular prion protein (PrP^C^)-forming amyloid fibrils that propagate by elongation and fission^1,2^. Prions exist as diverse strains enciphered by variation in fibril structure that cause distinct clinicopathological disease phenotypes^2^. Although prion diseases are transmissible conditions, the large majority of human prion disease actually occurs as a late-onset sporadic condition, sporadic Creutzfeldt–Jakob disease (CJD), and almost all other cases result from autosomal dominant coding mutations in the prion protein gene (*PRNP*), causing the inherited prion diseases. Acquired or iatrogenic CJD is rare, currently accounting for approximately 1% of recognized cases. Iatrogenic CJD arises from accidental inoculation with prions during medical or surgical procedures. These include former use of human cadaveric pituitary-derived growth hormone or gonadotrophin, dura mater and corneal grafting and via contaminated neurosurgical instruments. An epidemic human prion disease, kuru, occurred in Papua New Guinea and was transmitted by ingestion of human tissue at mortuary feasts as a mark of mourning and respect. Since the cessation of this practice in the late 1950s, kuru gradually disappeared but enabled documentation of the range of incubation periods of human prion infection; the mean incubation period is approximately 12 years but can exceed 50 years^3^. There is also worldwide genetic evidence for prehistoric human prion disease epidemics^4^. A novel human acquired prion disease, variant CJD, arose in the 1990s, following dietary exposure to the zoonotic prion disease of cattle, bovine spongiform encephalopathy (BSE)^5,6^.

The far wider relevance of prion mechanisms was first exemplified with the discovery of yeast prions^7^ but has also widened considerably with the recognition that the more common human neurodegenerative diseases, including Alzheimer’s and Parkinson’s diseases^8^, involve accumulation and spread of assemblies of misfolded host proteins in what is often described as a ‘prion-like’ fashion with experimental transmission of relevant pathology in primates^9^ or mouse models^10^. However, the importance for human disease was unclear until the recognition of human transmission of amyloid-beta (Aβ) pathology via iatrogenic routes after prolonged incubation periods, causing iatrogenic cerebral amyloid angiopathy (CAA) and raising the possibility that iatrogenic Alzheimer’s disease may occur at even longer latency^11,12^.

Between 1959 and 1985, at least 1,848 patients in the United Kingdom were treated with human cadaveric pituitary-derived growth hormone (c-hGH)^13^. Worldwide, over 200 cases of iatrogenic CJD have occurred as a consequence of childhood treatment with c-hGH^14^, with 80 cases recorded in the United Kingdom^15^. We first reported human-to-human transmission of Aβ pathology in people who had received c-hGH in childhood and died of iatrogenic CJD^11^; we later demonstrated that some of the archived batches of c-hGH used to treat these people contained measurable quantities of Aβ (and tau) and that this historical material still contained Aβ seeding activity able to transmit pathology to mice^12^. These experiments provided clear evidence that iatrogenic Aβ transmission had occurred in people treated with c-hGH. Multiple postmortem reports of iatrogenic Aβ transmission caused by c-hGH^16–18^ (and also via other routes^16,19–24^) were subsequently made by others.

The Aβ peptide is implicated in Alzheimer’s disease and is found in the form of parenchymal deposits, including neuritic plaques, and parenchymal and leptomeningeal vascular aggregation, corresponding to CAA. CAA is seen as a co-pathology in the large majority of people with Alzheimer’s disease and can also independently present with intracerebral hemorrhage^25^. There are now a number of clinical descriptions of iatrogenic CAA in people who developed symptoms during life^26^, typically due to brain hemorrhage. All affected individuals had prior exposure to cadaveric dura mater or had childhood neurosurgical procedures, both of which are recognized routes for prion transmission causing iatrogenic CJD^27^. However, until now, there have been, to our knowledge, no clinical (that is, premortem) descriptions of iatrogenic disease caused by Aβ transmission in c-hGH recipients, despite the substantial experimental evidence for transmission via this route.

## Further new clinical presentations in c-hGH recipients

The National Prion Clinic (NPC) forms part of the United Kingdom national referral system for suspected prion diseases and coordinates the National Prion Monitoring Cohort (NPMC), a longitudinal study of individuals with confirmed prion diseases (sporadic, inherited, iatrogenic or variant forms) and those at risk of inherited, iatrogenic or variant CJD^28^, including people previously treated with c-hGH^29^.

Since our earlier report of iatrogenic CAA in this cohort, eight further individuals with a history of treatment with c-hGH were referred to, or reviewed by, the NPC between 2017 and 2022. All individuals had received c-hGH prepared using the Wilhelmi or Hartree-modified Wilhelmi preparation (abbreviated here as HWP) method (Table 1), the preparation that has been implicated in all cases of iatrogenic CJD in the United Kingdom^13,29^. We previously reported^12^ values of Aβ-40, Aβ-42 and tau in HWP batches received by four of the individuals we report here (HWP 40, HWP 42, HWP 43, HWP 47 and HWP 51, received by cases 1, 5, 6 and 7) and demonstrated Aβ transmission in mice from two batches (HWP 42 and HWP 51) received by three of these individuals (cases 1, 5 and 7); these batches also resulted in Aβ transmission in certain patients in our previous description of patients who died of iatrogenic CJD^11,12^. The diagnosis of iatrogenic CJD was excluded in all eight individuals on the basis of clinical presentation, neuroimaging and biomarkers and, in two cases, by postmortem examination. Clinical descriptions of all cases are provided in the Supplementary Information.

**Table 1:** c-hGH received by each case

Case	Indication for c-hGH	c-hGH preparations received						
		HWP	FL	K	LJ	R	TPL	Other
1	Craniopharyngioma; secondary (postoperative) hypopituitarism	HWP 37, HWP 41, HWP 43, HWP 48, *HWP 51* Additional 7 ‘Wilhelmi’ batches received, batch IDs unknown	FL4, FL9	.	LJ0003, LJ0005, LJ0006	.	Four batches, batch IDs unknown	.
2	Craniopharyngioma; secondary (post-operative) hypopituitarism	12 ‘Wilhelmi’ batches received, batch IDs unknown	.	.	.	Two batches, batch IDs unknown	.	.
3	Silver–Russell Syndrome	HWP 8 Additional 6 ‘Wilhelmi’ batches received, batch IDs unknown	.	.	.	Five batches, batch IDs unknown	.	One batch ‘HGH’, further details unknown
4	Septo-optic dysplasia	HWP 5 (2×), HWP 7, HWP 8 (2×), HWP 11, HWP 19, HWP 24, HWP 28, HWP 32, HWP 39 Additional 3 ‘Wilhelmi’ batches received, batch IDs unknown	FL1, FL8, FL9, FL10	.	.	R17, R21 Additional ‘Raben’ batch received, batch ID unknown	.	.
5	Medulloblastoma; post-therapy growth hormone insufficiency	*HWP 42* (×2) Additional ‘HWP’ batch received, batch ID unknown	FL6	.	.	.	.	Three batches ‘HGH’, further details unknown
6	Isolated idiopathic GH deficiency	HWP 30 (2×), HWP 35 (2×), HWP 37, HWP 38, HWP 40, HWP 45, HWP 46, HWP 47 (2×), HWP 50 (2×)	FL2, FL3, FL5 (2×), FL11 (2×)	K79972	.	.	TPL5, TPL8, TPL9, TPL10	One batch ‘HGH’, further details unknown
7	Isolated idiopathic GH deficiency	HWP 44 (×2); HWP 50 (2×); *HWP 51* (2×)	FL7	K79972	LJ0004 (2×)	.	TPL3, TPL6	.
8	Craniopharyngioma; secondary (postoperative) hypopituitarism	HWP 00, HWP 37, HWP 39, HWP 41, HWP 50	FL5 (2×), FL7, FL8 (2×)	K79250	LJ0005 (2×)		TPL10, TPL14, TPL18, TPL24 Additional 5 ‘TPL’ batches received, batch IDs unknown	Two batches ‘HGH’, further details unknown

Case numbers refer to clinical descriptions in the Supplementary Information. Quantification of Aβ-40, Aβ-42 and Tau for underlined batches were reported by us previously^12^. Batches in italics have demonstrated Aβ transmission in mice.

c-hGH, cadaveric human growth hormone; FL, St. Bartholomew’s Hospital preparation (Roos–Lowry method); HWP, Hartree-modified Wilhelmi preparation; K, Kabi commercial preparation (Roos method); LJ, commercial preparation (Roos method); R, Raben preparation; TPL, Centre for Applied Microbiology and Research (CAMR) Porton Down preparation.

Five of these eight c-hGH recipients (Table 2; cases 2, 3, 4, 5 and 8) were referred with symptoms consistent with early-onset dementia, with progressive cognitive impairment in two or more domains severe enough to affect the performance of usual activities of daily living; in some cases, progression was rapid (Supplementary Information). Symptom onset was between the ages of 38 years and 49 years in four patients (cases 3, 4, 5 and 8) and at age 55 years in the remaining patient (case 2). In three of these five patients (cases 3, 4 and 8), a diagnosis of Alzheimer’s disease had been made before referral to the NPC; two individuals presented with typical amnestic symptoms (cases 4 and 8) and met National Institute on Aging and Alzheimer’s Association (NIA-AA) diagnostic criteria^30^ for probable Alzheimer’s disease, and the other individual (case 3) presented with with non-amnestic (language) symptoms. The remaining two patients met NIA-AA diagnostic criteria^30^ for probable Alzheimer’s disease with non-amnestic presentations (dysexecutive (case 2) and language (case 5)). All five cases would meet Diagnostic and Statistical Manual of Mental Disorders, Fifth Edition (DSM V) criteria for major neurocognitive disorder due to Alzheimer’s disease^31^. Of the remaining three individuals, one had symptoms (onset aged 42 years; case 1) meeting NIA-AA criteria for mild cognitive impairment^32^ (predominantly affecting behavior and personality); one had subjective cognitive symptoms only (case 7); the other was asymptomatic (case 6). For those with symptoms, the latency from c-hGH exposure was three to four decades (Table 2).

**Table 2:** Characteristics of all c-hGH recipients

	Age (years)												
Case	Symptom onset	Current	c-hGH treatment (range)	HWP treatment (range)	Latency from HWP, range (years)	Radiotherapy	Prior intellectual disability	Educational level	Living independently before symptom onset?	Criteria			*APOE* genotype
										NIA-AA	DSM-V	AT(N)	
1^a^	42	Deceased aged 47	4–13	4–9	33–38	Yes	No	University	Yes	MCI (dysexecutive)	No	Insufficient data	Data unavailable
2^a^	55	Deceased aged 57	11–17	11–17	38–44	Yes	No	Secondary School	Yes	Non-amnestic (dysexecutive)	Yes	No	ε3 / ε4
3	38	54	2–8	4–8	30–34	No	No	Secondary School	Yes	Non-amnestic (language)	Yes	Alzheimer’s disease	ε3 / ε3
4	46	56	6–14	6–13	33–40	No	Yes	Specialist school	Yes (with family support)	Amnestic	Yes	Insufficient data	Data unavailable
5	49	Deceased aged 54	11–12	11–12	37–38	Yes	Yes	Specialist school	Yes (with family support)	Non-amnestic (language)	Yes	Insufficient data	Data unavailable
6	N/A	57	12–17	12–15	N/A	No	No	Secondary School	Yes	No	No	Alzheimer’s disease	ε3 / ε3
7	N/A	56	14–16	14–16	N/A	No	No	Secondary School	Yes	No	No	No	ε2 / ε3
8	48	53	9–15	9–11	37–39	Yes	No	Secondary School	Yes	Amnestic	Yes	Alzheimer’s pathologic change	ε3 / ε3

Case numbers refer to clinical descriptions in the Supplementary Information.

^a^indicates recipients for whom pathological data were available.

APOE, apolipoprotein E gene; AT(N), amyloid tau neurodegeneration classification system for Alzheimer’s disease; c-hGH, human cadaver-derived pituitary growth hormone; CSF, cerebrospinal fluid; CT, computed tomography; DSM V, Diagnostic and Statistical Manual of Mental Disorders; HWP, Wilhelmi method (of c-hGH preparation); MCI, mild cognitive impairment; MRI, magnetic resonance imaging; N/A, not applicable; NIA-AA, National Institute on Aging-Alzheimer’s Association; PET, positron emission tomography.

## Investigative findings

**Fig. 1:**
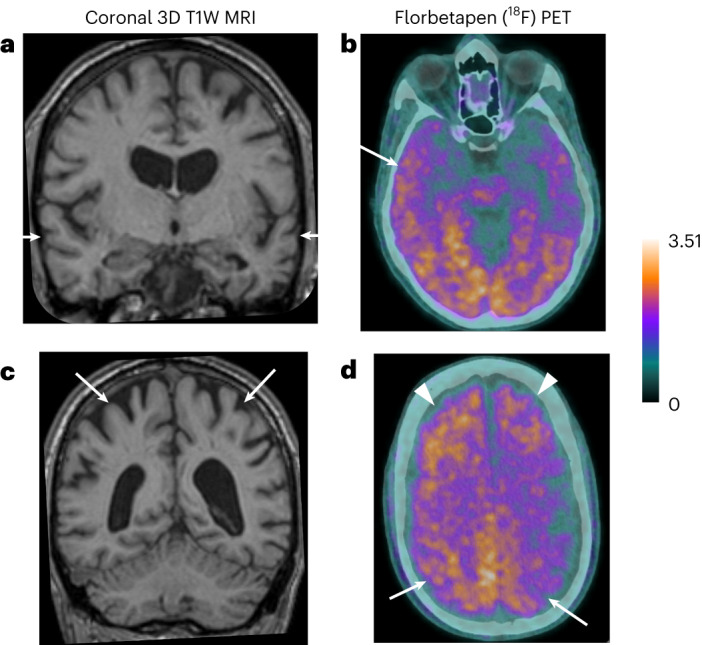
Magnetic resonance and amyloid-PET (^18^F-Florbetapen) images—case 3. **a**, High-resolution three-dimensional (3D) T1-weighted (T1W) magnetic resonance (MR) coronal image through the temporal lobes demonstrates volume loss within the temporal lobes bilaterally (arrows) and also marked central atrophy. **b**, Axial PET images demonstrate diffuse increased tracer uptake in the cortex and subcortical white matter, increased in the right temporal lobe compared to the left. **c**, High-resolution MR (3D T1W) coronal image through superior parietal lobules bilaterally demonstrates marked volume loss (arrows). **d**, Axial PET images demonstrate marked tracer uptake within the superior parietal lobules bilaterally (arrows) in addition to increased uptake in the bilateral frontal lobes (arrowheads).

**Fig. 2:**
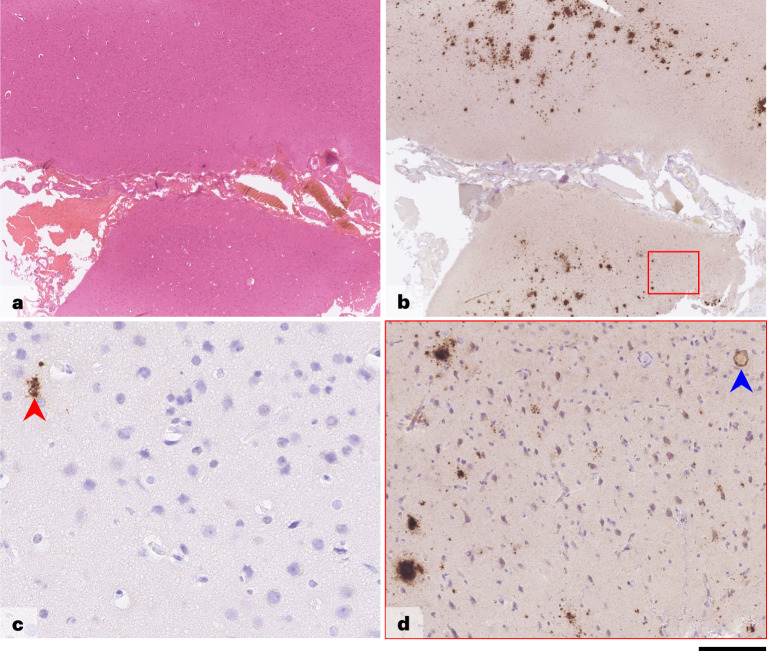
Brain biopsy—case 2. Images shown are from a left frontal lobe brain biopsy. H&E-stained preparation (**a**) shows full-thickness well-preserved cortical hexa-laminar cytoarchitecture with unremarkable overlying leptomeninges. Immunostaining for Aβ (**b** and **d**) shows frequent diffuse parenchymal deposits with no plaques with central amyloid cores and a single blood vessel with concentric Aβ angiopathy but no associated inflammation. Hyperphosphorylated tau (**c**) is restricted to rare dystrophic deposits, with no evidence of neuronal or glial tau pathology. Brain postmortem findings are provided in the Supplementary Information. Scale bar, 750 µm in **a** and **b**, 50 µm in **c** and 100 µm in **d**. Aβ antibody: clone 6F3D, dilution 1:50, source DAKO, product number M0872. Hyperphosphorylated tau antibody: clone AT8, dilution 1:1,200, source Invitrogen (Thermo Fisher Scientific), product number MN1020.

**Fig. 3:**
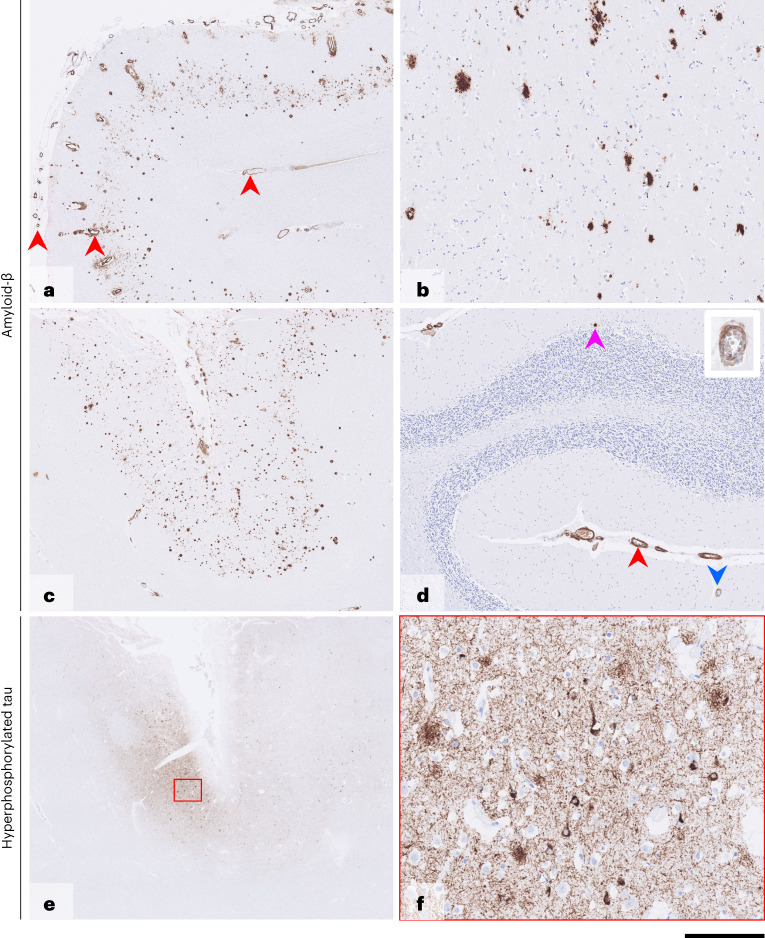
Postmortem brain tissue—case 1. Immunostaining for Aβ (**a**–**d**) shows frequent parenchymal deposits in the cortex (**a** and **c**) and caudate nucleus (**b**), with rare, isolated deposits in the cerebellar cortex (**d**, pink arrowhead). In the cerebrum (**a** and **c**), there is widespread, concentric amyloid angiopathy in the leptomeninges, cortex and subcortical white matter (red arrowheads in **a**), and, in the cerebellum (**d**), there is widespread concentric amyloid angiopathy in the leptomeninges (red arrowhead) and occasionally in the cerebellar cortex (blue arrowhead; inset shows vessel at higher magnification), without associated inflammation. Immunostaining for hyperphosphorylated tau (AT8) of the insular cortex (**e** and **f**) shows pan-cortical patches of a dense meshwork of neuropil threads, frequent pre-tangles, occasional tangles and moderately frequent neuritic plaques. Scale bar, 1.5 mm in **a**, 250 µm in **b**, 170 µm in **c**, 400 µm in **d**, 1.8 mm in **e** and 130 µm in **f**. Aβ antibody: clone 6F3D, dilution 1:50, source DAKO, product number M0872. Hyperphosphorylated tau antibody: clone AT8, dilution 1:1,200, source Invitrogen (Thermo Fisher Scientific), product number MN1020.

**Extended Data Fig. 1:**
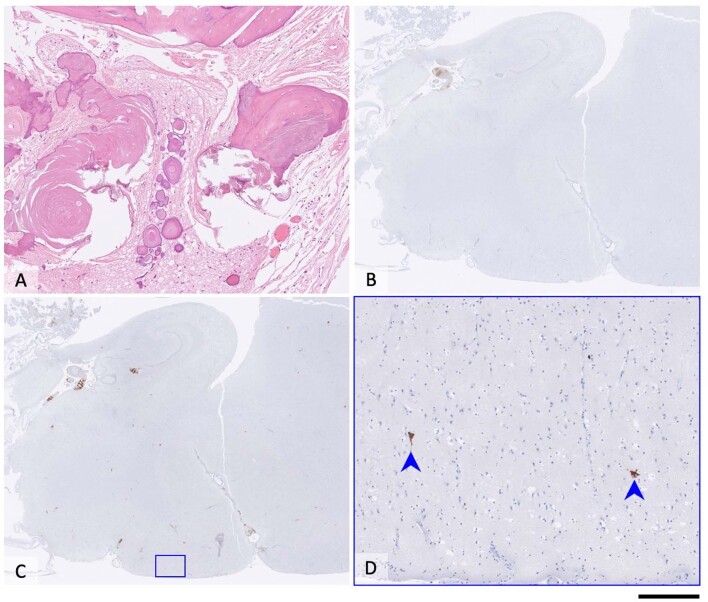
Post-mortem brain tissue, Case 2. Haematoxylin and eosin-stained preparation (**a**) demonstrates extensively calcified and ossified adamantinomatous craniopharyngioma invading into the brain tissue, without any histological signs of malignant transformation. Immunostaining for amyloid-β shows no evidence of pathology presence in the hippocampal region and parahippocampal gyrus (**b**) or basal ganglia, brainstem and cerebellum (not shown). Hyperphosphorylated tau pathology in the limbic region is restricted to rare isolated neurofibrillary tangles, pre-tangles and threads in the entorhinal cortex (**c** and **d**, with blue arrowheads in D). Scale bar: 230µm in A; 3mm in B and **c**, and 220µm in **d**. Amyloid-β antibody: clone 6F3D, dilution 1:50, source DAKO, product number M0872. Hyperphosphorylated tau antibody: clone AT8, dilution 1:1200, source Invitrogen (Thermo), product number MN1020.

Given these observations, we reviewed relevant investigations completed as part of the standard clinical care that these patients had received. Two patients clinically diagnosed with Alzheimer’s disease before our review—one amnestic (case 8) and one non-amnestic (case 3)—had biomarker changes compatible with the diagnosis, meeting the amyloid/tau/neurodegeneration, or AT(N), criteria for disorders within the Alzheimer’s continuum (Fig. 1 and Table 2)^32^. The other patient (case 4) with amnestic Alzheimer’s disease did not have molecular biomarker studies performed but did show progressive volume loss on sequential brain imaging (computed tomography (CT)), which involved the mesial temporal, frontal and parietal lobes bilaterally, consistent with a neurodegenerative process and not accounted for by another process (their underlying diagnosis of septo-optic dysplasia, or radiotherapy, which they had never received). Of the other two patients presenting with non-amnestic Alzheimer’s disease, one (case 2) had elevation of cerebrospinal fluid (CSF) total tau and phosphorylated tau; brain-restricted postmortem examination showed non-specific Aβ (diffuse deposits with patchy distribution restricted to the neocortex; single cortical blood vessel with concentric mural Aβ deposition) and tau deposition not meeting pathological criteria for Alzheimer’s disease (Fig. 2, Supplementary Information and Extended Data Fig. 1). The other patient with non-amnestic Alzheimer’s disease (case 5) did not have molecular biomarker studies during life but showed progressive bi-frontal atrophy on sequential magnetic resonance imaging (MRI) scans in addition to cerebellar post-surgical changes. The person with mild cognitive impairment (case 1) was not investigated for this during life; postmortem examination of several brain regions (neocortex, basal ganglia and cerebellum; Fig. 3) showed widespread Aβ deposition (equivalent to Thal^33^ stage 5 and CERAD^34^ score 2) as well as a focus of Alzheimer’s type neurofibrillary tangle, pre-tangle and a dense thread pathology with moderately frequent neuritic plaques in the insular cortex. There was also widespread, severe cerebral Aβ angiopathy affecting many of the blood vessels in the cerebral and cerebellar leptomeninges, cerebral cortex, cerebral subcortical white matter and occasional vessels in the cerebellar cortex, with focal capillary involvement in the cerebral cortex. The individual with subjective cognitive symptoms (case 7) had a solitary right temporal cerebral microbleed, with no evidence of atrophy or other features of cerebral small vessel disease and normal biomarkers (amyloid positron emission tomography (PET) and CSF studies). The asymptomatic individual met the AT(N) criteria for Alzheimer’s disease (reduced CSF Aβ-42/Aβ-40 ratio 0.053, where values less than 0.065 are suggestive of fibrillar Aβ deposition; elevated CSF phospho-tau 181, 64 pg ml^−1^; normal range, 0–58 pg ml^−1^).

Genetic testing for causative variants associated with adult-onset neurodegenerative disorders was negative for five of eight cases (samples unavailable for cases 1, 4 and 5). One patient (case 2) was heterozygous for a variant of unknown significance in the amyloid precursor protein gene (*APP*) (NM_000484.3:c.1486A>C; p.Lys496Gln); this is a rare, likely benign, variant^35,36^. The panel of genes tested is provided in the Methods; *APOE* (apolipoprotein E) genotypes are provided in Table 2; only one patient (case 2) carried an ε4 allele. We additionally reviewed other risk genes associated with Alzheimer’s disease (*ABCA7*, *SORL1* and *TREM2)*, and no relevant variants were identified.

The c-hGH recipients that we report here have developed new and progressive disturbances of cognition that meet standard definitions for dementia (five cases) or mild cognitive impairment (one case); they also show changes consistent with Alzheimer’s disease (definite in four cases; suggestive in two patients with a clinical diagnosis of dementia). Their relatively young age makes sporadic Alzheimer’s disease unlikely^37,38^, and, as inherited causes have been excluded, we considered that their symptoms and biomarker findings are a consequence of Aβ transmission from contaminated c-hGH received in childhood. Iatrogenic Aβ transmission has resulted in human disease on several occasions, with iatrogenic CAA now a recognized cause of early-onset stroke^26^, and the individuals whom we describe in this report have received c-hGH batches that contain quantifiable Aβ and can be used to transmit Aβ experimentally in a new host^12^.

## Consideration of alternative explanations for these findings

First, we considered whether childhood intellectual disability, occurring in our cases as either a consequence of neoplasia treatment or underlying congenital diagnosis, might explain these findings; intellectual disability has been associated with a higher prevalence of dementia with onset at earlier ages^39–43^. However, only two of the patients whom we describe had an intellectual disability from childhood. Second, we considered whether the underlying diagnosis causing growth hormone deficiency might have resulted in their adult cognitive symptoms. We did not find any published association among craniopharyngioma, Russell–Silver syndrome, septo-optic dysplasia or medulloblastoma and either Alzheimer’s disease or Aβ pathology in humans, apart from in cases of iatrogenic Aβ transmission, as already reported. Third, we considered whether growth hormone deficiency itself might explain our findings; growth hormone has effects on brain structure and cognition in both children and adults^44–46^. We do not consider it plausible that growth hormone deficiency could explain the marked and, in some cases, rapid cognitive deterioration experienced by these patients, all of whom had maintained their (adult) level of functioning for decades. Any hypothetical growth hormone deficiency persisting from childhood would have existed throughout this period of normal cognition and independent living. Moreover, growth hormone deficiency cannot explain the biomarker profiles observed. Furthermore, we did not identify any published reports describing an association between growth hormone deficiency and Alzheimer’s disease or other Aβ pathology, apart from cases of iatrogenic Aβ transmission. Finally, we considered the effect of cranial radiotherapy, which was used as a treatment in four of the patients whom we describe. Radiotherapy treatment in adults with primary and metastatic brain tumors has been associated with mild cognitive impairment and dementia^47^, although not Alzheimer’s disease specifically^48^; data on adult survivors of childhood brain tumors are limited^49^. We identified one postmortem study^50^ reporting increased Aβ deposition in adults of equivalent age (30–59 years) with adult-onset malignancy (extracranial primary tumors) but without dementia. In the series of patients that we describe here, Aβ deposition was more marked in those treated with radiotherapy. However, we do not consider it plausible that radiotherapy can explain our findings. Aβ deposition after radiotherapy is likely a response to acute radiation injury; a similar process occurs after traumatic brain injury^51,52^ in which Aβ rapidly accumulates in the acute phase and then clears over a period of days^53–56^. Data from the above report^50^ show that the mean survival time to death in individuals with Aβ deposition (70 d; range, 10–180 d) is shorter than that in the group without Aβ deposition (120 d; range, 30–300 d). This finding supports the argument that Aβ deposits after radiotherapy are cleared with time, as is the case in traumatic brain injury, although there are no specific data to confirm or refute this hypothesis. We found no other published association between radiotherapy and Alzheimer’s disease or other Aβ pathology. The temporal correlation between onset of cognitive symptoms and radiotherapy treatment in our cases argues against the latter mediating these former, and two of our symptomatic cases did not receive radiotherapy at all.

## Association with the HWP preparation of c-hGH

As detailed earlier, the NPC is part of the United Kingdom national referral service for individuals with all forms of prion disease, including those ‘at risk’ for developing prion disease (including c-hGH recipients). Those who develop neurological or cognitive symptoms are routinely discussed with and referred to our service. Given our national referral role, our case ascertainment is very high. In this report, we have provided details for every c-hGH recipient discussed with, or referred to, our service since our earlier report^12^. All c-hGH recipients were treated with c-hGH prepared using multiple different methods; however, notably, all patients described here and in our previous reports^11,12^ received c-hGH prepared by the HWP method. We previously showed^12^ that HWP batches uniformly contain significant levels of Aβ contamination in distinction to batches prepared by other methods, which were uniformly negative. Such archived HWP c-hGH samples were also used to transmit Aβ pathology to mice^12^. No patients who have only been treated with non-HWP c-hGH have been referred to our service. There is no evidence that HWP was preferentially administered for particular underlying diagnoses^13^, and details of the preparations that a patient has received are established from archival records after referral to us; referring clinicians are unaware of which preparations were used, and so the absence of referrals to the NPC cannot reflect bias from the referring clinician. Together, this strongly suggests that the clinical phenotypes that we report here are caused by HWP c-hGH. Although we cannot exclude the possibility that childhood diagnosis and/or its treatment might modify the risk of developing cognitive symptoms, if these childhood diagnoses were alone responsible for the observed findings, we would have expected equivalent referrals of patients who had received only non-HWP c-hGH, which we did not receive.

The data presented here were collected during the provision of routine clinical care, and there are, therefore, inevitable differences in how patients were investigated, with consequent variation in the clinical, pathological, genetic and biomarker data available. Although we do not have genetic data for three of the patients (cases 1, 4 and 5), these patients had no family history of early-onset dementia (or stroke) to suggest a familial form of Alzheimer’s disease. We are also unable to comment on risk variants in these cases, but these alone are unlikely to fully explain the phenotype (including age of onset) observed. For example, although the *APOE* ε4 genotype can be associated with an earlier age of onset, this is still in the 60s for homozygotes^57^.

Taken together, the only factor common to all of the patients whom we describe is treatment with the HWP subtype of c-hGH. Given the strong experimental evidence for Aβ transmission from relevant archived HWP c-hGH batches, we conclude that this is the most plausible explanation for the findings observed. The clinical syndrome developed by these individuals can, therefore, be termed iatrogenic Alzheimer’s disease, and Alzheimer’s disease should now be recognized as a potentially transmissible disorder.

## Phenotypic considerations in iatrogenic Alzheimer’s disease

Perhaps unsurprisingly, these patients differ phenotypically from patients with sporadic and familial Alzheimer’s disease. For prion diseases, it is long recognized that acquired forms of human prion disease differ in clinical presentation, progression and neuropathological features from sporadic and inherited forms of prion disease and that these, in turn, are different from one another. It is notable that acquired prion diseases associated with peripheral exposure to prions—for example, iatrogenic CJD from c-hGH inoculation (intramuscular injection) and kuru (ingestion)—are generally associated with a cerebellar onset with early ataxia and a more prolonged clinical course than typical sporadic CJD or iatrogenic CJD associated with direct central nervous system exposure to prions (for example, after neurosurgery or corneal grafting^58^), which usually present with cognitive symptoms. Notably, amyloid precursor protein (APP)-transgenic mice develop different patterns of pathology after peripheral (intraperitoneal) inoculation of Aβ seeds when compared either to intracerebral inoculation or to their later-onset spontaneous pathology phenotype^59,60^. In our cases, the early involvement of multiple cognitive domains is not typical of sporadic late-onset Alzheimer’s disease. Our cases are also atypical for inherited Alzheimer’s disease, which usually presents amnestically but can differ from sporadic Alzheimer’s disease in having earlier symptom onset and early additional neurological features (such as myoclonus, seizures, spastic paraparesis, cerebellar and extrapyramidal signs) as well as atypical cognitive presentations, including behavioral, dysexecutive or language symptoms^61,62^. These cases also differ from individuals diagnosed with iatrogenic CAA (for example, due to exposure to cadaveric dura mater^26^), who have generally presented with one or more intracerebral hemorrhages and have other structural imaging markers seen in sporadic CAA. By contrast, the patients whom we describe had progressive cognitive symptoms, sometimes over a decade, with unusually young age at onset and with very limited evidence of CAA or other cerebral small vessel disease on brain imaging.

## A possible role for Aβ strains

Another contributor to the differences between iatrogenic Alzheimer’s disease and other types of Alzheimer’s disease might be the presence of Aβ strains. In prion diseases, strain type is a key determinant of disease phenotype, and sporadic, iatrogenic and variant CJD, kuru and inherited prion diseases all involve multiple prion strains^63^. Prion strains produce distinct disease phenotypes that persist on serial passage in laboratory animals; this protein-based inheritance is encoded by differences in prion protein folding and glycosylation^1,2,64^. Furthermore, prion strains exist as a ‘cloud’ or quasispecies with diverse structures, such that strain adaptation can occur in a new host with a different prion protein sequence or under drug selection by agents binding to the dominant strain species^1,64^. Structural investigations of Aβ from distinct clinical subtypes of Alzheimer’s disease using solid-state nuclear magnetic resonance^65^ and cryogenic electron microscopy^66^ provide early supportive evidence, and a biological basis, for Aβ strains.

Strain type might also explain why clinical CAA (characterized by symptomatic and asymptomatic cerebral hemorrhagic events) seems less prevalent in c-hGH recipients. CAA is observed at autopsy in individuals with clinical CAA but also in the large majority of individuals with pathologically defined Alzheimer’s disease. Individuals with Alzheimer’s disease tend not to have the hemorrhagic presentations of CAA, although imaging features can be present (cerebral microbleeds)^67^. A postmortem report^17^ including c-hGH recipients without iatrogenic CJD found pathological CAA in the two oldest individuals (aged 42 years and 45 years); data on brain imaging were not provided. In our patients, who lived for longer periods after c-hGH exposure than in our original report^11^, pathological data were available for only two patients (cases 1 and 2), one of whom did have widespread, severe CAA. For the remainder, four had appropriate MRI (that is, with sequences allowing identification of structural imaging markers associated with CAA), with only equivocal evidence for clinical CAA. We hypothesize that iatrogenic Aβ amyloidosis caused by c-hGH can result in a different clinical phenotype (possibly mediated by Aβ strain type), in which clinical CAA is less prominent (although CAA may be present pathologically, as in sporadic Alzheimer’s disease). Additionally, it is entirely possible that some iatrogenic cases of Alzheimer’s disease may differ markedly from sporadic and inherited forms in both clinical and neuropathological features; the full spectrum of dementias caused by Aβ transmission remains to be elucidated.

## Other factors contributing to phenotypic diversity

Our cases as a group demonstrate diverse clinical presentations and investigative findings; not all were symptomatic and not all fully meet the current diagnostic criteria for sporadic Alzheimer’s disease. As described above, this is to be expected and is likely to reflect clinical features inherent to iatrogenic aetiology. It is important to recognize that these patients were treated for different durations of time, at different stages of maturity, with different quantities of HWP c-hGH (Tables 1 and 2) and with each HWP batch containing variable amounts of Aβ seeds. Each patient will also have a unique combination of as yet unidentified host factors that confer susceptibility to and/or protection from Aβ transmission. Together, these are likely to contribute to the diversity in phenotype observed at the individual level. We hope and expect that our observations will stimulate reports of similar cases by others, as was the case after our initial description of iatrogenic CAA^11,16–24^, so that the full clinical and pathological phenotype of iatrogenic Alzheimer’s disease can be better understood.

## Discussion

Although Alzheimer’s disease arises predominantly as a sporadic condition of late adult life, there are rarer early-onset Mendelian forms caused by mutations in the *APP* gene or in genes (*PSEN1* and *PSEN2*) known to alter its enzymatic cleavage. We now provide evidence that Alzheimer’s disease is also transmissible in certain circumstances and, therefore, that Alzheimer’s disease (like Aβ-CAA) has the full triad of etiologies (sporadic, inherited and rare acquired forms) characteristic of conventional prion diseases. This should further emphasize that the principles of prion biology have relevance for other neurodegenerative diseases involving the accumulation of diverse assemblies of misfolded host proteins, which may have propagating and neurotoxic forms^1,68^. Our cases suggest that, similarly to what is observed in human prion diseases, iatrogenic forms of Alzheimer’s disease differ phenotypically from sporadic and inherited forms, with some individuals remaining asymptomatic despite exposure to Aβ seeds due to protective factors that, at present, are unknown.

Our previous report of transmission of Aβ pathology, causing the disease iatrogenic CAA, led to international meetings to consider public health risk assessment and risk management^69,70^. It is important to emphasize that the cases described here developed symptoms after repeated exposure to contaminated c-hGH, over a period of years, and that treatment with c-hGH was discontinued many years ago (in the United Kingdom, in 1985); there is no evidence that Aβ can be transmitted in other contexts—for example, during activities of daily life or provision of routine care. The individuals whom we previously reported with iatrogenic CAA had died from iatrogenic CJD after exposure to c-hGH contaminated with both CJD prions and Aβ seeds (and also tau). Given the far higher population prevalence of Alzheimer’s pathology than CJD, it is expected that c-hGH batches, prepared from very large pools of cadaveric pituitary glands, will be much more frequently contaminated by Aβ seeds than CJD prions. Consequently, we considered the possibility that some c-hGH-exposed individuals who did not develop CJD might progress to develop the full pathological features of Alzheimer’s disease at even longer incubation periods than those we described for iatrogenic CAA. The symptomatic cases that we report here are consistent with that conclusion and should prompt both further consideration of public health implications and the primary prevention of iatrogenic Alzheimer’s disease—for example, by ensuring effective decontamination of surgical instruments. Additionally, the extent to which prion-like mechanisms are involved in Alzheimer’s pathogenesis may have important bearings on therapeutic strategies targeting disease-related Aβ assemblies if these exist as quasispecies and show strain diversity and propagation kinetics akin to conventional prions with a diversity of propagating and/or neurotoxic conformers^1,65,68,71–73^. Structurally diverse conformers, present as minor components, may be selected for propagation by a drug that binds to the dominant species, potentially leading to the development of resistance.

## Methods

Data presented here were collected during the provision of routine clinical care and have been de-identified to prevent patient identification. Analyses were conducted in compliance with all relevant ethical regulations; additional details, where applicable, are provided in the sections below.

### Brain biopsy and postmortem brain tissue preparation

Informed consent to use the tissue for research was obtained from the next of kin, and ethical approval was obtained from the local research ethics committee of the UCL Queen Square Institute of Neurology.

The biopsy sample (case 2) was collected as part of routine clinical care, in accordance with standardized local neurosurgical protocols. Autopsies were carried out in a postmortem room designated for high-risk autopsies. Postmortem tissues were extensively sampled from multiple brain regions.

Tissue samples were immersed in 10% buffered formalin, and potential prion infectivity was inactivated by immersion into 98% formic acid for 1 h, followed by further fixation in formalin and processing to paraffin wax. Tissue sections were routinely stained with hematoxylin and eosin (H&E), followed by immunostaining with anti-PrP ICSM35 (D-Gen Ltd., 1:1,000), anti-phospho-tau (AT8 Invitrogen (Thermo Fisher Scientific), 1:12,00) and anti-bA4 (DAKO, 6F3D, 1:50). Immunostaining was performed on a Ventana Discovery automated immunohistochemical staining platform (Roche), following the manufacturer’s guidelines, using biotinylated secondary antibodies and an HRP-conjugated streptavidin complex and diaminobenzidine as a chromogen.

### Genetic testing

Informed written consent for genetic testing was obtained for each patient. Next-generation sequencing (NGS) was performed commercially by CENTOGENE (https://www.centogene.com/). CentoXome Solo Genomic DNA is enzymatically fragmented, and target regions are enriched using DNA capture probes. These regions include approximately 41 Mb of the human coding exome (targeting >98% of the coding RefSeq from the human genome build GRCh37/hg19) as well as the mitochondrial genome. The generated library is sequenced on an Illumina platform to obtain at least 20× coverage depth for more than 98% of the targeted bases. An in-house bioinformatics pipeline, including read alignment to GRCh37/hg19 genome assembly and revised Cambridge Reference Sequence (rCRS) of the Human Mitochondrial DNA (NC_012920), variant calling, annotation and comprehensive variant filtering, is applied. All variants with minor allele frequency (MAF) of less than 1% in the gnomAD database and disease-causing variants reported in HGMD, in ClinVar or in CentoMD are evaluated. The investigation for relevant variants is focused on coding exons and flanking ±10 intronic nucleotides of genes with clear gene–phenotype evidence (based on OMIM information). All potential patterns for mode of inheritance are considered. In addition, provided family history and clinical information are used to evaluate identified variants with respect to their pathogenicity and disease causality. Variants are categorized into five classes (pathogenic, likely pathogenic, variants of unknown significance (VUS), likely benign and benign) in accordance with American College of Medical Genetics and Genomics (ACMG) guidelines for classification of variants. All relevant variants related to the phenotype of the patient are reported. CENTOGENE has established stringent quality criteria and validation processes for variants detected by NGS. Variants with low sequencing quality and/or unclear zygosity are confirmed by orthogonal methods. Consequently, a specificity of more than 99.9% for all reported variants is warranted. Mitochondrial variants are reported for heteroplasmy levels of 15% or higher. The copy number variation (CNV) detection software has a sensitivity of more than 95% for all homozygous/hemizygous and mitochondrial deletions as well as heterozygous deletions/duplications and homozygous/hemizygous duplications spanning at least three consecutive exons. For the uniparental disomy (UPD) screening, a specific algorithm is used to assess the well-known clinically relevant chromosomal regions (6q24, 7, 11p15.5, 14q32, 15q11q13, 20q13 and 20).

Variants (including copy number variants) in the following genes associated with adult-onset neurodegeneration were reviewed: C9orf72, ATXN2, PRNP, ABCA7, ALS2, ANG, ANXA11, APOE, APP, ARSA, ATL1, ATP7B, BSCL2, CCNF, CHCHD10, CHMP2B, CP, CSF1R, CYLD, CYP27A1, DCTN1, ERBB4, EWSR1, FIG4, FTL, FUS, GLE1, GRN, HEXA, HNRNPA1, HNRNPA2B1, HSPD1, ITM2B, KIF5A, MAPT, MATR3, MT-ATP6, MT-ATP8, MT-CO1, MT-CO2, MT-CO3, MT-CYB, MT-ND1, MT-ND2, MT-ND3, MT-ND4, MT-ND4L, MT-ND5, MT-ND6, MT-RNR1, MT-RNR2, MT-TA, MT-TC, MT-TD, MT-TE, MT-TF, MT-TG, MT-TH, MT-TI, MT-TK, MT-TL1, MT-TL2, MT-TM, MT-TN, MT-TP, MT-TQ, MT-TR, MT-TS1, MT-TS2, MT-TT, MT-TV, MT-TW, MT-TY, NEFH, NEK1, NOTCH3, NPC1, OPTN, PANK2, PFN1, PRPH, PSEN1, PSEN2, REEP1, SETX, SIGMAR1, SLC52A3, SNCA, SOD1, SORL1, SPAST, SPG11, SQSTM1, TAF15, TARDBP, TBK1, TFG, TREM2, TUBA4A, TYROBP, UBE3A, UBQLN2, VAPB, VCP and WASHC5. The CentoXome analysis does not include repeat expansions.

### Reporting summary

Further information on research design is available in the Nature Portfolio Reporting Summary linked to this article.

## Online content

Any methods, additional references, Nature Portfolio reporting summaries, source data, extended data, supplementary information, acknowledgements, peer review information; details of author contributions and competing interests; and statements of data and code availability are available at 10.1038/s41591-023-02729-2.

### Supplementary information

Supplementary Results (case descriptions).

Reporting Summary

## Figures and Tables

**Extended Data Table 1:**
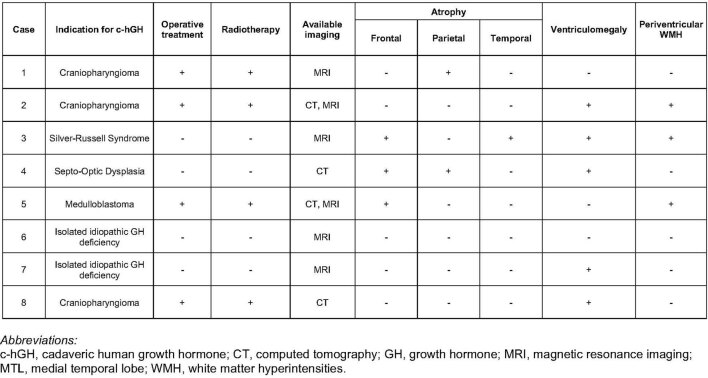
Structural brain imaging in c-hGH recipients

## Data Availability

All available de-identified clinical data generated or analyzed during this study are included in this published article and its Supplementary Information files. Patient identifiable information, including genetic data, cannot be made publicly available for reasons of patient privacy and confidentiality but are available from the corresponding author upon reasonable request with supporting ethical approval.
