# Supplementary material for: Iatrogenic Alzheimer’s disease in recipients of cadaveric pituitary-derived growth hormone
Source: Nat Med. 2024 Jan 29;30(2):394–402. doi: 10.1038/s41591-023-02729-2 (PMC10878974; doi:10.1038/s41591-023-02729-2)
Supplement: Supplementary file 1 — Supplementary Results (case descriptions). [file 41591_2023_2729_MOESM1_ESM.pdf]

nature medicine

Article

https://doi.org/10.1038/s41591-023-02729-2

Iatrogenic Alzheimer's disease in recipients of cadaveric pituitary-derived growth hormone

In the format provided by the authors and unedited

SUPPLEMENTARY MATERIAL

Supplementary Results

Case Descriptions

Case 1

A 47-year-old man was unexpectedly found dead at home (2019).

His past medical history in childhood was notable for sub-total surgical resection of a partially cystic intra- and suprasellar craniopharyngioma aged 3 years (1974; dura closed without patch). Aged 5 years (1976), he had tumour recurrence; this was again treated operatively (subtotal resection of solid component and evacuation of cystic component, via left frontal craniotomy; dura closed without patch) and subsequently with post-operative radiotherapy.

This person developed partial hypopituitarism following his first surgery (with evidence of secondary adrenal insufficiency, secondary hypothyroidism, and total growth hormone deficiency), in addition to hypothalamic dysfunction (diabetes insipidus). He received treatment with c-hGH between 1976 and 1985, including c-hGH isolated by the Hartree-modified Wilhelmi procedure (HWP). He otherwise made a good post-operative recovery, although he remained partially sighted and was slightly less co-ordinated than his peers throughout his life. His past medical history was otherwise unremarkable. He attained his developmental milestones at the appropriate times and did well at school, initially attending catering college and later university (to study computer science and business studies). He worked for a software start-up company and later as a civil servant; he lived independently and had an active social life.

He remained well until late 2014 (aged 42 years), when his vision deteriorated; he first noticed that he was unable to make sense of letters on his computer screen at work. This continued to progress, until large parts of his visual field were “greyed out”. He was found to have recurrence of his tumour, which was treated with further debulking surgery (February 2015) and stereotactic radiosurgery (April 2015), after which his vision in the right eye improved slightly. In October 2016 the vision in his right eye deteriorated again; repeat imaging in early 2017 demonstrated enhancement in the right cavernous sinus and hypothalamus, which was treated with further stereotactic radiosurgery in August 2017 with evidence of treatment response. He was additionally noted to have a progressive third nerve palsy on the right from the autumn of 2016, with a worsening ptosis evident from August 2017. In January 2019 he was noted to be bumping into things on the left, and repeat imaging demonstrated further growth of the recurrent tumour.

Throughout this period (i.e. from late 2014, from the age of 42 years), he became more withdrawn and apathetic, and required prompting from family members for tasks that he previously would have performed, for example those relating to personal hygiene, tidying his home and managing his own affairs (such as opening his post). Although he had always had a “sweet tooth”, this became more apparent,

and he was noted to be eating substantially more sweet and "junk" foods. His family also noticed some subtle social changes, for example that he would wear his shoes inside the house, and that he would start his meal at the dinner table before other people seated were ready, both of which were unusual for him. His mood seemed lower than usual, and he was no longer able to engage in social or leisure activities that he previously enjoyed due to his worsening visual impairment. His memory, language and behaviour were otherwise normal, and he continued to work as a volunteer at a local charity shop. His support worker would visit him once a week, primarily to help him with shopping given his visual impairment.

There was no family history of stroke, dementia or other neurological disease.

As the death was unexplained, a post-mortem examination was carried out locally by Her Majesty's Coroner in order to ascertain cause of death. This was given as malignant transformation of craniopharyngioma; a small deposit of tumour was identified anterior to the midbrain, with features suggestive of (but not diagnostic for) a craniopharyngioma with malignant transformation. There was no evidence of intracerebral haemorrhage, although assessment of the brain was limited by extensive traumatic removal artefact of the brain, which included complete disruption of medial temporal lobes, the right cerebellar hemisphere and left occipital lobe.

Post-mortem examination of the brain additionally identified florid amyloid-β pathology. Parenchymal amyloid-β deposits in the form of diffuse deposits were seen with widespread distribution across the brain, including neocortical regions, the caudate nucleus and putamen and as scanty minute deposits also in the cerebellar cortex, corresponding to Thal phase 5. In the neocortex (left frontal, and left and right temporal cortical regions), there were moderately frequent plaques with central amyloid cores, corresponding to CERAD score 2. There was also widespread, severe cerebral amyloid-β angiopathy affecting many of the blood vessels in the cerebral and cerebellar leptomeninges, cerebral cortex, cerebral subcortical white matter, and occasional vessels in the cerebellar cortex, with focal capillary involvement in the cerebral cortex. Cerebral amyloid angiopathy associated inflammation was not observed in any of the regions. Furthermore, with patchy distribution in the insular cortex, there was pan-cortical Alzheimer's type neurofibrillary tangle, pre-tangle and a dense thread pathology with moderately frequent neuritic plaques. The regions required for assessment of Braak stages 1 to 3 (that is, medial temporal lobe regions) were unavailable, and therefore it was not possible to determine these Braak stages. The absence of tau pathology in frontal and temporal regions suggests that tau pathology would not be consistent with Braak stages 4 to 6. There was no evidence of misfolded prion protein pathology, α-synucleinopathy or TDP43 proteinopathy in the available examined cortical, deep grey nuclei or cerebellar regions.

Case 2

A 55-year-old woman (2016) developed new onset behavioural change, confusion and ataxia. This first manifested when she failed to collect her usual prescriptions from her family doctor, and her family found that she could become aggressive and

argumentative for no obvious reason. Two months after this started, she was admitted to hospital with a suspected Addisonian crisis, at which point her confusion had worsened. She was discharged home but readmitted shortly afterwards with confusion, intermittent drowsiness and deterioration in her mobility. In the months after this, she experienced a steady deterioration in her memory and gait, which became increasingly unsteady. She also developed visual hallucinations, some of which were complex (a dead cousin; a fruit machine) and odd beliefs, for example that she had attended a party and been on holiday, when this was not the case. Clinical examination identified significant visual impairment with limited upgaze, a mild left-sided hemiparesis associated with brisk reflexes and an extensor plantar on that side, all of which were longstanding. Her gait was noted to be ataxic, and she was disoriented in time and place.

Her childhood past medical history was notable for sub-total surgical resection of a suprasellar craniopharyngioma aged 5 years (1966); no dural patch was used. Post-operatively she developed seizures (treated with phenytoin), partial hypopituitarism (with evidence of secondary adrenal insufficiency and secondary hypothyroidism) and hypothalamic dysfunction (diabetes insipidus). She received treatment with c-hGH between the ages of 11 and 17 (1973 to 1978), including HWP batches. She did well at school, often coming top of her class and in the “top set” for most subjects (highest ability range); she later qualified as a nurse.

CT imaging aged 21 (1982) showed evidence of tumour recurrence, but not to the extent that surgical intervention was necessary. Aged 28 years (1989) she developed drowsiness and ataxia and was found to have papilloedema; CT imaging is reported as showing further tumour progression, for which she had another sub-total resection and later a ventriculo-peritoneal shunt. Three years later (1992), aged 31 years, her vision deteriorated again and an MRI confirmed further tumour recurrence, which was managed non-operatively with radiotherapy. She had to stop working as a nurse shortly after this, due to visual impairment and a left sided hemiparesis. She lived independently, and shared a home with her parents.

Both parents developed dementia at an old age (80s) and were living at the time of her deterioration (she had been caring for them both at home, prior to becoming unwell). Her two younger brothers were both alive and well.

CSF analysis for neurodegenerative markers demonstrated normal amyloid-β 1-42 (1222 pg/mL; normal range 627 to 1322 pg/mL), elevated total tau (954 pg/mL; normal range 146 to 595 pg/mL) and phosphorylated tau (81 pg/mL; normal range 24 to 68 pg/mL). Standard CSF PrP RT-QuIC was positive; 14-3-3 was negative and S100b were normal.

In view of the clinical presentation, stable brain imaging, positive RT-QuIC result and ongoing uncertainty about the underlying diagnosis, a left frontal brain biopsy was performed (May 2017). A drain was inserted into the cystic lesion at the same time.

The brain biopsy (Figure 2, main manuscript) demonstrated frequent diffuse parenchymal amyloid-β deposits without central cores in the cortex, but only a single cortical blood vessel with concentric amyloid-β deposition in the wall, with no evidence of leptomeningeal cerebral amyloid angiopathy or any signs of

inflammation. Hyperphosphorylated tau pathology was restricted to rare dystrophic deposits in the neuropil, with no evidence of neuronal or glial inclusions. The biopsy showed well-preserved hexa-laminar cortical cytoarchitecture, with no evidence of disease-associated prion protein, α-synuclein or TDP43 pathology and no other specific pathology.

She developed SIADH post-operatively and had a single generalised tonic-clonic seizure (managed with levetiracetam). There was some clinical improvement following drainage of the cystic lesion, but approximately six months after this she was readmitted with drowsiness and hypernatraemia. Repeat imaging demonstrated stable ventricular configuration and further neurosurgical intervention was deemed inappropriate given her clinical condition.

She continued to progress rapidly, with worsening cognition (particularly affecting memory and orientation) and gait ataxia. Within 15 months of first symptom onset, she could utter single words (short sentences at best), required a pureed diet, and was confined to bed; she was noted to have finger myoclonus. A few months later (18 months after initial symptom onset), she was minimally conscious and required enteral tube feeding; she died shortly thereafter.

Brain restricted post-mortem examination (Extended Data Figure 1) demonstrated an extensively calcified craniopharyngioma with widespread invasion into surrounding brain tissue. Analysis of neocortical areas included left frontal (superior and middle frontal gyri), temporal (superior and middle temporal gyri), parietal (superior and inferior parietal lobules) and occipital (striate and peristriate) cortical regions. Amyloid-β pathology in the form of diffuse deposits with patchy distribution was restricted to the neocortex (Thal phase 1, CERAD score 0). Detailed examination of the brain showed no further evidence of amyloid angiopathy in the cerebral or cerebellar parenchyma or leptomeninges. Hyperphosphorylated tau pathology in addition to exceedingly rare isolated dystrophic deposits in the cortex, was restricted to rare neurofibrillary tangles and pre-tangles, and very rare threads in the entorhinal cortex. This limited tau pathology would not meet the requirements for Braak stage 1. There was no evidence of disease-related prion protein, TDP43 or α-synuclein pathology or any other specific pathology.

<u>Case 3</u>

A 48-year-old man was referred to a Cognitive Disorders Clinic with gradually progressive memory disturbance, dysarthria, anomic aphasia, gait and co-ordination difficulties and personality change. Approximately 10 years prior to referral (aged approximately 38 years), he noticed that he was increasingly forgetful and was having difficulties communicating; at this time he was working as a senior manager, living alone and managing his affairs independently. Five years prior to referral, aged approximately 43 years, he was no longer able to live alone; his sister increasingly had to manage his financial and personal affairs, and he became socially withdrawn.

He was known to have Silver-Russell Syndrome, which was diagnosed shortly after birth. Silver-Russell Syndrome is characterised by prenatal and postnatal growth retardation, as well as a number of other features, including feeding difficulties,

craniofacial features, body asymmetry and other skeletal abnormalities[1,2]. Treatment with growth hormone is used to increase adult height, as well as for its effects on appetite and muscle mass[1], and this patient received c-hGH treatment between the ages of 3 and 9 years (1971 to 1977), including HWP batches. He was treated for meningococcal meningitis aged 2 months, but had no other medical issues in childhood. He attended a mixed ability special needs school and did not demonstrate any developmental delay or learning disability. His past medical history was otherwise notable for hyperthyroidism (diagnosed aged 47 years; treated with radio-iodine); he was also a carrier for haemochromatosis (*HFE* gene). There was no relevant family history.

Formal neuropsychological testing demonstrated severe intellectual decline compared to premorbid estimates of function, with profound expressive and receptive language difficulties and near global impairment across multiple domains, including naming, memory, executive function and processing speed. His MRI at this time demonstrated mild left temporal and left hippocampal atrophy. CSF analysis demonstrated low amyloid-β 1-42 (298 pg/mL; normal range 627 to 1322 pg/mL) and elevated total tau (671 pg/mL; normal range 146 to 595 pg/mL). The S100 was normal; 14-3-3 and PrP RT-QuIC were negative. Amyloid-PET (using 18F-Florbetaben; Figure 1, main manuscript) confirmed widespread amyloid-β deposition in the frontal, parietal and temporal lobes (right more than left).

In view of his investigative findings, a diagnosis of young-onset Alzheimer's disease was made; in the absence of any other (i.e. genetic) cause, this was felt to be iatrogenic and relate to his previous c-hGH exposure. Treatment with donepezil was started, and memantine added approximately 6 months later. He developed mood disturbances approximately one year after referral, which responded well to sertraline; he was noted to have marked apraxia and finger myoclonus at this time. He has remained under routine review of the Cognitive Disorders Clinic (now five years since original referral, and 15 years since first symptom onset), with evidence of slow but continued progression of his cognitive symptoms.

<u>Case 4</u>

A 49-year-old woman was referred to her local Cognitive Disorders Clinic with progressive memory and language difficulties and behavioural change from the age of 46 years. Her mother had noticed that she had started to misplace objects such as her glasses, credits cards and shoes, and become disoriented in familiar locations, including inside a car; she was also speaking less than usual, with new word finding difficulties and her spelling became newly abnormal. Her dietary preferences changed, with new avoidance of sweet foods, and she developed repetitive socially inappropriate behaviours (such as picking her nose, and at her skin and pubic hair in public) and agitation. These changes were associated with significant weight loss and increased somnolence. These symptoms progressed over three years, by which stage she was finding it difficult to care for herself or hold a meaningful conversation, at which point she had to stop living at home and move into a nursing home. Approximately four years after symptom onset, she was still able to walk, but unable to care for herself (feeding, dressing) and was incontinent; by this time, she had become completely non-verbal.

Her past medical history was notable for septo-optic dysplasia, diagnosed aged 2 years when she was noted to have significant visual impairment and failure to thrive. Septo-optic dysplasia is a congenital disorder characterised by optic nerve hypoplasia, agenesis of midline brain structures and hypoplasia of the hypothalamic–pituitary axis, with resulting hypopituitarism[3]. Neurological involvement occurs in around 70% of cases and often co-exists in those with craniofacial features; this can manifest as epilepsy (30% of patients), spastic paraparesis, and developmental delay (motor and intellectual). This patient received treatment with c-hGH, including HWP preparations, between 1973 and 1981. She attended a specialist school for children with visual impairment, and although not formally diagnosed at the time, was likely to have mild learning difficulties. She had a job working in a facility for people with learning difficulties, which would involve tasks such as packing, sorting or assembly; she lived with her parents, but was able to travel to her local town independently and take care of herself. She developed generalised tonic-clonic seizures aged 18 years, which were well managed with carbamazepine and only recurred on occasions where carbamazepine withdrawal was attempted.

Neurological examination approximately four years following symptom onset demonstrated a longstanding visual impairment, with no pupillary response to light in either eye and no reliable response to visual threat. There was a tremor of the left upper limb but no obvious ataxia; her gait was noted to be slow and hesitant, with some ignition failure.

A clinical diagnosis of young onset Alzheimer's disease was made on the basis of her CT head imaging, which demonstrated generalised atrophy, including that involving the medial temporal lobes, which was far in advance of that expected given her age.

Case 5

A 49-year-old woman developed an unsteady and shuffling gait; this progressed such that she had falls and required a walking frame to mobilise. She was noted to have developed a tremor of both hands and a tendency to lean to the right at a similar time. Approximately five years after these symptoms originally started, aged 53 years, she started to experience a change in her cognitive function, and was noted to be intermittently confused. This deteriorated rapidly and began to involve her speech, which was noted to taper off, and eventually progressed such that she would often only say three or four words. At this stage she was noted to have insomnia, hallucinations and "bobbing" movements of her trunk; she was no longer able to care for herself. These symptoms were attributed to a urinary tract infection, and showed some initial improvement with treatment before deteriorating once again. Approximately 8 months after this, aged 54 years, things deteriorated to such an extent that she required admission to hospital, where she again was noted to be hallucinating and experiencing significant insomnia. She also developed difficulties in swallowing, such that she required a feeding via gastrostomy, became bedbound and was noted to have myoclonus. Blood tests at this stage demonstrated renal failure, severe hypercalcaemia and hypokalaemia, and treatment of these abnormalities resulted in a marked (but not total) recovery. Over the next 8 months

or so, with management of her hypercalcaemia (the aetiology of which remains unknown) her symptoms improved significantly; she was once again able to speak in full sentences and swallow solid food, stand and transfer with assistance, and even mobilise a few steps with a frame at her best. She had no further hallucinations and her insomnia also improved. However, she still had marked difficulties in comprehension and with her short-term memory, and her speech had not completely returned to normal, with her family reporting use of nonsense words at times. She continued to live at home with her father, but was now fully dependent for all aspects of self care.

Aged 3 years, this patient was diagnosed with a medulloblastoma; this was managed surgically, and with post-operative radiotherapy and chemotherapy; she later required a ventriculo-peritoneal shunt. She was noted to have some intellectual disability thereafter, and attended a specialist school, where she learned to speak but not to read or write. At the age of 10 years she was involved in a serious road traffic accident in which she fractured her femur, and she developed epilepsy shortly after this, which was well controlled on medication. Her growth was noted to be restricted and she was treated with c-hGH from the age of 11 years (between 1978 and 1980), including batches produced by the HWP method. After leaving school she lived with her parents and could care for herself independently, but did not work. In her late 20s she had further seizures, which once again were well controlled with medication, and migraine.

There was no relevant family history of note. Her mother died aged 58 years (cause of death not known to us); her father and siblings (one sister and two brothers) are in good health.

Clinical examination at the time of her first hospital admission (five years after symptom onset; aged 54 years) demonstrated longstanding reductions of visual acuity and restriction of her visual fields. There was evidence of a squint (right eye deviated downwards and outwards), but no ptosis, and restriction of adduction and vertical movements of the right eye , with inconsistent upbeat nystagmus. She had a mild dysarthria but normal cough and tongue movements. In the upper limbs, she had mild postural tremor and evidence of ataxia bilaterally, the latter more evident on the right. In the lower limbs, there was mild ataxia on heel-shin testing. In all limbs, there was mild weakness proximally but good power distally. All reflexes were present but not brisk; plantars were extensor. The grasp reflex was absent. From a cognitive perspective, she was able to obey simple commands; she was oriented in place but not in time. She was able to recognise a number of silhouetted drawings of animals and birds, and could name her sister. Further cognitive examination was not possible.

Serial brain imaging with MRI demonstrated progressive bi-frontal atrophy in addition to cerebellar post-surgical changes.

This person died following an acute illness at the age of 54.

Case 6

A 54-year-old man was enrolled as a research participant in the National Prion Monitoring Cohort (NPMC). They received treatment with c-hGH for isolated idiopathic growth hormone deficiency. This is a longitudinal cohort study of people with a confirmed diagnosis of prion disease, or at risk of developing a prion disease, and involves regular (usually annual) clinical assessments and investigations. This participant has remained asymptomatic in the two years since his enrolment. He received c-hGH between 1977 and 1983, including HWP preparations. His past medical history was otherwise notable for anxiety and depression.

Formal neuropsychological testing demonstrated a normal cognitive profile, with evidence of anxiety.

Brain imaging demonstrated normal intracranial appearances. CSF analysis for neurodegenerative markers initially demonstrated low-normal amyloid-β 1-42 (628pg/mL; normal range 627 to 1322 pg/mL, normal total tau (345 pg/mL; normal range 146 to 595 pg/mL) and elevated phosphorylated Tau 181 (64 pg/mL; normal range 0 to 58 pg/mL). Repeat testing aged 56 years demonstrated a CSF amyloid-β 1-42 / 1-40 of 0.053, ratio consistent with fibrillar amyloid-β deposition in the brain (ratio < 0.065).

Case 7

A 52-year-old man was referred with symptoms in keeping with subjective cognitive disturbance, including fluctuating navigational difficulties and impaired recall of recent events, and episodes of acute confusion. They received treatment with c-hGH for isolated idiopathic growth hormone deficiency. He enrolled in the NPMC and has been under review for four years, and has remained clinically stable during this period. He received c-hGH between 1980 and 1983, including HWP. His past medical history is otherwise notable for epilepsy (initially in childhood, with recurrence in adulthood), an episode of meningococcal meningitis aged 18 years and hypertension.

Formal neuropsychological assessment demonstrated cognitive performance within normal limits, with impairment on a single test of executive function (the remaining tests of executive function being normal) in the context of symptoms consistent with severe anxiety and moderate depression. His cognitive performance has remained stable on annual assessment.

Brain MRI demonstrated a solitary right temporal microhaemorrhage, with no evidence of atrophy or other features of cerebral small vessel disease. Amyloid-PET using the ligand 18F-Florbetapen only demonstrated physiological tracer distribution in the white matter . CSF analyses showed normal levels of neurodegenerative markers, including CSF amyloid-β 1-42, amyloid-β 1-42/1-40 ratio and total tau, and negative PrP RT-QuIC. These CSF measures have remained stable on annual assessment.

Case 8

A 48-year-old man developed subtle symptoms of poor memory and cognitive slowing. These gradually progressed, and he lost his job approximately two years after these symptoms first occurred, likely as a consequence of his cognitive symptoms. At around this time, he was admitted to hospital after becoming seriously unwell with an Addisonian crisis and sepsis, and his memory difficulties significantly worsened after this period of ill health. These continued to progress over the following three years, and have particularly declined in the past year (approximately five years after these symptoms were first noticed). He is frequently confused over times and dates, and can misremember situations; examples include thinking he is late for interviews which are not scheduled, and believing that his deceased mother is still alive. He has occasionally mistaken his wife for his sister, and often asks questions repetitively. He has also lost interest in activities he previously enjoyed, such as golf, and he is eating more sweet foods than previously. Despite these memory impairments, he remains able to travel locally independently, does not mislay objects and can use familiar objects such as the TV remote control (although sometimes he struggles with the keypad of his new mobile phone).

His past medical history is notable for a craniopharyngioma, diagnosed when he was aged 6 years, which was removed surgically and treated subsequently with radiotherapy. He received treatment with c-hGH between the ages of 9 and 15 years (1979 to 1985) including HWP preparations. He was otherwise well as a child, completed schooling at 16 years and then worked in a number of retail businesses until he became more recently unwell (as described). His past medical history is otherwise notable for testicular lymphoma (diagnosed four years after onset of his cognitive symptoms), and for which he is now receiving active treatment.

His paternal aunt developed dementia in her 70s; there is no known family history of stroke, dementia or other neurological disease.

On examination of the cranial nerves, there was evidence of a mild left ptosis and a subtle dysarthria. Limb examination revealed a symmetrical, very mild tremor on finger nose testing, with no evidence of bradykinesia. There was no evidence of apraxia and the Luria test of sequential movements was normal. In the lower limbs, heel-shin co-ordination was mildly impaired bilaterally, and there was mild loss of vibration appreciation at the left ankle; Romberg test was negative. Reflexes were brisker on the left throughout; the left plantar was extensor. The remainder of the neurological examination, including examination of gait, was unremarkable. Cognitive examination demonstrated an MMSE score of 25/30, with loss of points on orientation, recall and concentration.

Formal neuropsychological testing demonstrated performances below expectations across multiple domains, including processing speed, attention, auditory working memory, language and executive functions. The assessor also noted “various frontal-type behaviours” exhibited throughout the assessment, including impulsivity, distractibility, impatience and an apparent lack of performance anxiety. Repeat neuropsychological testing approximately two years later showed severe under-functioning, which was more pronounced in the non-verbal domain. Focal testing

revealed striking memory and executive dysfunction; confrontation naming, synonym matching and irregular word-reading were also poor. These findings were felt to be consistent with pronounced fronto-temporal compromise.

CSF analyses demonstrated a slightly reduced amyloid-β 1-42/1-40 ratio (0.062; values less than 0.065 suggestive of abnormal amyloid-β deposition); CSF tau was in the normal range. PrP RT-QuIC was negative. EEG was within normal limits; CT imaging of the head demonstrated stable appearances compared to the prior scan completed two years previously with evidence of a previous right frontal craniotomy and associated right frontal atrophy, residual calcified suprasellar tumour, calcification of the basal ganglia bilaterally and diffuse cerebellar atrophy. Repeat CSF testing, performed approximately 18 months after the previous lumbar puncture, showed an amyloid-β 1-42/1-40 ratio of 0.083, total tau 295 pg/mL (normal range 146 to 595 pg/mL) and phospho-tau 181 level of 43 pg/mL (normal range 0 - 58 pg/mL).

References

1 Wakeling, E. L. *et al.* Diagnosis and management of Silver-Russell syndrome: first international consensus statement. *Nat Rev Endocrinol* **13**, 105-124, doi:10.1038/nrendo.2016.138 (2017).

2 Giabicani, E., Netchine, I. & Brioude, F. New clinical and molecular insights into Silver-Russell syndrome. *Curr Opin Pediatr* **28**, 529-535, doi:10.1097/MOP.0000000000000379 (2016).

3 Sataite, I., Cudlip, S., Jayamohan, J. & Ganau, M. Septo-optic dysplasia. *Handb Clin Neurol* **181**, 51-64, doi:10.1016/B978-0-12-820683-6.00005-1 (2021).

4 Purro, S. A. *et al.* Transmission of amyloid-beta protein pathology from cadaveric pituitary growth hormone. *Nature* **564**, 415-419, doi:10.1038/s41586-018-0790-y (2018).
