# Supplementary material for: Iatrogenic Alzheimer’s disease in recipients of cadaveric pituitary-derived growth hormone
Source: Nat Med. 2024 Jan 29;30(2):394–402. doi: 10.1038/s41591-023-02729-2 (PMC10878974; doi:10.1038/s41591-023-02729-2)
Supplement: Supplementary file 2 — Reporting Summary [file 41591_2023_2729_MOESM2_ESM.pdf]

nature portfolio

Corresponding author(s): John Collinge

Last updated by author(s): 7th November 2023

Reporting Summary

Nature Portfolio wishes to improve the reproducibility of the work that we publish. This form provides structure for consistency and transparency in reporting. For further information on Nature Portfolio policies, see our Editorial Policies and the Editorial Policy Checklist.

Statistics

For all statistical analyses, confirm that the following items are present in the figure legend, table legend, main text, or Methods section.

n/a	Confirmed	
☒	☐	The exact sample size (*n*) for each experimental group/condition, given as a discrete number and unit of measurement
☒	☐	A statement on whether measurements were taken from distinct samples or whether the same sample was measured repeatedly
☒	☐	The statistical test(s) used AND whether they are one- or two-sided<br>*Only common tests should be described solely by name; describe more complex techniques in the Methods section.*
☒	☐	A description of all covariates tested
☒	☐	A description of any assumptions or corrections, such as tests of normality and adjustment for multiple comparisons
☒	☐	A full description of the statistical parameters including central tendency (e.g. means) or other basic estimates (e.g. regression coefficient) AND variation (e.g. standard deviation) or associated estimates of uncertainty (e.g. confidence intervals)
☒	☐	For null hypothesis testing, the test statistic (e.g. *F*, *t*, *r*) with confidence intervals, effect sizes, degrees of freedom and *P* value noted<br>*Give P values as exact values whenever suitable.*
☒	☐	For Bayesian analysis, information on the choice of priors and Markov chain Monte Carlo settings
☒	☐	For hierarchical and complex designs, identification of the appropriate level for tests and full reporting of outcomes
☒	☐	Estimates of effect sizes (e.g. Cohen's *d*, Pearson's *r*), indicating how they were calculated

Our web collection on statistics for biologists contains articles on many of the points above.

Software and code

Policy information about availability of computer code

Data collection	No software was used.
Data analysis	Genetic testing was performed by an external commercial company (Centogene; https://www.centogene.com), using in-house software. All available information is included in the manuscript.

For manuscripts utilizing custom algorithms or software that are central to the research but not yet described in published literature, software must be made available to editors and reviewers. We strongly encourage code deposition in a community repository (e.g. GitHub). See the Nature Portfolio guidelines for submitting code & software for further information.

Data

Policy information about availability of data

All manuscripts must include a data availability statement. This statement should provide the following information, where applicable:

- Accession codes, unique identifiers, or web links for publicly available datasets
- A description of any restrictions on data availability
- For clinical datasets or third party data, please ensure that the statement adheres to our policy

All available de-identified clinical data generated or analysed during this study are included in this published article and its supplementary information files. Patient identifiable information, including genetic data, can not be made publicly available for reasons of patient privacy and confidentiality, but are available from the corresponding author on reasonable request with supporting ethical approval

Research involving human participants, their data, or biological material

Policy information about studies with human participants or human data. See also policy information about sex, gender (identity/presentation), and sexual orientation and race, ethnicity and racism.

Reporting on sex and gender	Sequential patients presenting to National Prion Clinic who had been treated with c-hGH were included. Full details of each case are provided in the Supplementary Material.
Reporting on race, ethnicity, or other socially relevant groupings	Sequential patients presenting to National Prion Clinic who had been treated with c-hGH were included. Full details of each case are provided in the Supplementary Material.
Population characteristics	Covariate analyses not performed. This was not a pre-designed research study - the manuscript instead describes a series of cases presenting to our service. Full details of each case are provided in the Supplementary Material.
Recruitment	Not applicable. No participants were recruited. This was not a pre-designed research study. The paper is a description of sequential cases presenting to our clinical service.
Ethics oversight	Data presented were collected during the provision of routine clinical care, and have been de-identified to prevent patient identification. Analyses were conducted in compliance with all relevant ethical regulations.

Note that full information on the approval of the study protocol must also be provided in the manuscript.

Field-specific reporting

Please select the one below that is the best fit for your research. If you are not sure, read the appropriate sections before making your selection.

- [x] Life sciences
- [ ] Behavioural & social sciences
- [ ] Ecological, evolutionary & environmental sciences

For a reference copy of the document with all sections, see nature.com/documents/nr-reporting-summary-flat.pdf

Life sciences study design

All studies must disclose on these points even when the disclosure is negative.

Sample size	Not applicable. This was not a pre-designed research study. Sample calculations were therefore not performed. The paper is a description of sequential cases presenting to our clinical service.
Data exclusions	Not applicable - no exclusions. The paper is a description of sequential cases presenting to our clinical service.
Replication	Not applicable - no experiments were performed. Figures show diagnostic histopathological investigations acquired in the course of routine clinical care.
Randomization	Not applicable - no intervention. The paper is a description of sequential cases presenting to our clinical service.
Blinding	Not applicable. The paper is a description of sequential cases presenting to our clinical service

Reporting for specific materials, systems and methods

We require information from authors about some types of materials, experimental systems and methods used in many studies. Here, indicate whether each material, system or method listed is relevant to your study. If you are not sure if a list item applies to your research, read the appropriate section before selecting a response.

Materials & experimental systems

n/a	Involved in the study	
☐	☒	Antibodies
☒	☐	Eukaryotic cell lines
☒	☐	Palaeontology and archaeology
☒	☐	Animals and other organisms
☐	☒	Clinical data
☒	☐	Dual use research of concern
☒	☐	Plants

Methods

n/a	Involved in the study	
☒	☐	ChIP-seq
☒	☐	Flow cytometry
☐	☒	MRI-based neuroimaging

Antibodies

Antibodies used	anti-bA4 DAKO 6F3D Catalogue #: M0872<br>anti-phospho-tau AT8 Invitrogen (Thermo) Catalogue #: MN102<br>Anti-PrP ICSM35 (D-Gen Ltd) - catalogue number not available
Validation	Highly characterised antibodies involved in many publications.<br>Anti-PrP ICSM35 (D-Gen Ltd) - PMID 18657254, PMID 16099923<br>anti-phospho-tau AT8 Invitrogen (Thermo) - PMID 7624036<br>anti-bA4 DAKO 6F3D - https://www.alzforum.org/antibodies/amyloid-v-6f3d

Clinical data

Policy information about clinical studies

All manuscripts should comply with the ICMJE guidelines for publication of clinical research and a completed CONSORT checklist must be included with all submissions.

Clinical trial registration	Not applicable - not a clinical trial.
Study protocol	Not applicable - not a pre-designed research study.
Data collection	Data presented were collected during the provision of routine clinical care.
Outcomes	Data presented were collected during the provision of routine clinical care. Outcomes to date are presented in the main manuscript, with full details in the Supplementary Material.

Magnetic resonance imaging

Experimental design

Design type	N/A MRI was standard clinical diagnostic imaging
Design specifications	*Specify the number of blocks, trials or experimental units per session and/or subject, and specify the length of each trial or block (if trials are blocked) and interval between trials.*
Behavioral performance measures	*State number and/or type of variables recorded (e.g. correct button press, response time) and what statistics were used to establish that the subjects were performing the task as expected (e.g. mean, range, and/or standard deviation across subjects).*

Acquisition

Imaging type(s)	*Specify: functional, structural, diffusion, perfusion.*
Field strength	*Specify in Tesla*
Sequence & imaging parameters	*Specify the pulse sequence type (gradient echo, spin echo, etc.), imaging type (EPI, spiral, etc.), field of view, matrix size, slice thickness, orientation and TE/TR/flip angle.*
Area of acquisition	*State whether a whole brain scan was used OR define the area of acquisition, describing how the region was determined.*

Diffusion MRI ☐ Used ☐ Not used

Preprocessing

Preprocessing software	*Provide detail on software version and revision number and on specific parameters (model/functions, brain extraction, segmentation, smoothing kernel size, etc.).*
Normalization	*If data were normalized/standardized, describe the approach(es): specify linear or non-linear and define image types used for transformation OR indicate that data were not normalized and explain rationale for lack of normalization.*
Normalization template	*Describe the template used for normalization/transformation, specifying subject space or group standardized space (e.g. original Talairach, MNI305, ICBM152) OR indicate that the data were not normalized.*
Noise and artifact removal	*Describe your procedure(s) for artifact and structured noise removal, specifying motion parameters, tissue signals and physiological signals (heart rate, respiration).*
Volume censoring	*Define your software and/or method and criteria for volume censoring, and state the extent of such censoring.*

Statistical modeling & inference

Model type and settings: *Specify type (mass univariate, multivariate, RSA, predictive, etc.) and describe essential details of the model at the first and second levels (e.g. fixed, random or mixed effects; drift or auto-correlation).*

Effect(s) tested: *Define precise effect in terms of the task or stimulus conditions instead of psychological concepts and indicate whether ANOVA or factorial designs were used.*

Specify type of analysis: ☐ Whole brain ☐ ROI-based ☐ Both

Statistic type for inference (See Eklund et al. 2016): *Specify voxel-wise or cluster-wise and report all relevant parameters for cluster-wise methods.*

Correction: *Describe the type of correction and how it is obtained for multiple comparisons (e.g. FWE, FDR, permutation or Monte Carlo).*

Models & analysis

n/a	Involved in the study
☒	☐ Functional and/or effective connectivity
☒	☐ Graph analysis
☒	☐ Multivariate modeling or predictive analysis
